# Self-reported cancer family history is a useful tool for identification of individuals at risk of hereditary cancer predisposition syndrome at primary care centers in middle-income settings: a longitudinal study

**DOI:** 10.1590/1678-4685-GMB-2014-0362

**Published:** 2016-06-03

**Authors:** Milena Flória-Santos, Luís Carlos Lopes-Júnior, Larissa de Melo Alvarenga, Mayara Segundo Ribeiro, Victor Evangelista de Faria Ferraz, Lucila Castanheira Nascimento, Gabriela Pereira-da-Silva

**Affiliations:** 1 Departamento de Enfermagem Materno-Infantil e Saúde Pública, Escola de Enfermagem de Ribeirão Preto, Universidade de São Paulo, Ribeirão Preto, SP, Brazil; 2 Departamento de Genética, Faculdade de Medicina Ribeirão Preto, Universidade de São Paulo, Ribeirão Preto, SP, Brazil

**Keywords:** Genetic predisposition to disease, Risk factors, Neoplasms, Family, Pedigree

## Abstract

Analysis of cancer family history (CFH) offers a low-cost genetic tool to identify familial cancer predisposition. In middle-income settings, the scarcity of individual records and database-linked records hinders the assessment of self-reported CFH consistency as an indicator of familial cancer predisposition. We used self-reported CFH to identify those families at risk for hereditary cancer syndromes in community-based primary care centers of a low-income Brazilian area. We also evaluated the consistency of the information collected by reassessing CFH five years later. We interviewed 390 families and constructed their pedigrees for genetic cancer risk assessment. We found 125 families affected by cancer, 35.2% with moderate to high risk of familial susceptibility to cancer, a number that represents a relatively high prevalence of potential hereditary cancer syndromes in the overall study sample. Upon reassessment of CFH in 14/20 families that were previously identified as having at least one first-degree and one second-degree relative affected by cancer, and presented moderate to high risk for developing cancer, 90% of initial pedigrees were confirmed. These results demonstrate the reliability of self-reports as a means of early identification of healthy individuals at risk, encouraging the wider use of this method in low- and middle-income primary care settings.

## Introduction

Family history is essential for identifying individuals at increased risk for primary and secondary cancers who could benefit from referral to genetics services (Weitzel *et al.*, 2011; Lu *et al.*, 2014). Identification of those individuals is crucial for early diagnosis, family management, and preventive care (Wood *et al.*, 2014), reducing cancer morbi-mortality and health system costs (Rubinstein *et al.*, 2011; Teng; Acheson, 2014).

The analysis of cancer family history (CFH) offers a low-cost, non-invasive genetic tool to track and diagnose familial cancer predisposition (Plat *et al.*, 2009; Valdez *et al.*, 2010; Janssens *et al.*, 2012). Indeed, CFH has been shown to be a strong predictor of disease risk and yet is significantly underused in primary care settings (Doerr and Teng, 2012; Teng and Acheson, 2014).

Reliance on self-reported CFH for use in clinical practice, decision-making regarding surveillance recommendations and the design of preventive interventions depends, however, on knowledge about the reliability and accuracy of this tool. Commonly, medical records, death certificates, and information obtained from cancer registries have been used to determine the accuracy of the self-reported cancer family history (Kelly *et al.*, 2007; Qureshi *et al.*, 2009). However, individual records and database-linked records are rarely available in low- and middle-income settings, making it difficult to establish whether self-reported CFH is a reliable indicator of familial cancer predisposition in these populations (Gomy and Diz, 2013).

In this study we used self-reported family history data to identify those families at risk for hereditary cancer syndromes at five community-based primary care centers in a middle-income area in Brazil. We also sought to evaluate the consistency of the information collected in spontaneous interviews by replicating the data collection five years later. Our results should encourage the wider use of self-reported cancer family history in low- and middle-income primary care settings.

## Subjects and Methods

We conducted a longitudinal study divided into two phases. In the first phase (started in 2008), a total of 410 families were selected through simple random sampling among 3,780 families attended at the five community-based primary care centers located in Ribeirão Preto, São Paulo, Brazil. The sample size was calculated considering an expected prevalence of 50% of CFH, and using a 95% confidence interval with α set at 5% considering a likely sampling loss of 15% (Pagano and Gauvreau, 2004).

A research team composed of five research assistants was trained by the first author to visit the families and collect their self-reported family history, with the purpose of selecting families at potential risk for hereditary cancer syndromes. We interviewed one informant per household, who was available at home, volunteered to participate in the study after listening to the research purposes, and then signed the consent form. The informant answered a questionnaire that included variables previously described in the literature (Feerro *et al.*, 2008; Lu *et al.*, 2014), such as personal and/or family history of cancer, degrees of relationship among family members affected by malignancies, gender, age, vital status, age at cancer onset, and type of primary cancer. Data were entered into Progeny pedigree-drawing software (Progeny Software, LLC, Indianapolis, IN, USA), and Statistical Package for the Social Sciences version 17.0 (SPSS Inc., Chicago, Illinois, USA) was used for descriptive statistical analysis.

All pedigrees were analyzed independently by two geneticists, a physician (VEFF), and a nurse (MFS) with expertise in oncogenetics, to perform genetic cancer risk assessment. In case of discordance regarding CFH evaluation, a third geneticist was consulted until consensus was reached, to ascertain data quality. We applied internationally established criteria to classify CFH in sporadic, familial, and hereditary cases, as well as to classify genetic cancer risk as low, moderate, and high (Table 1) (Schneider, 2002; Lindor *et al.*, 2008; Valdez *et al.*, 2010; NCCN, 2014; Vieira *et al.*, 2015).

**Table 1:** Criteria for cancer family history and genetic cancer risk classification.

Cancer family history	Criteria	Genetic cancer risk
Hereditary	At least one first and one second-degree relative affected by cancer	High
	Three or more family members with same or related cancers	
	Exhibit classical cancers of hereditary cancer syndromes	
	Majority of the cases exhibit an autosomal dominant pattern of inheritance	
	Multiple primary cancers in an individual	
	Presence of rare cancers	
	Excess of bilateral cancers	
	Presence of nonmalignant features previously associated with hereditary cancer syndromes	
	At least one relative diagnosed at a younger than usual age	
Familial	More cases of cancer within a family than statistically expected	Moderate
	More distant affected family members	
	Does not often exhibit classical features of hereditary cancer syndromes	
	Familial cancer clusters without a specific inheritance pattern	
	Variable age of onset	
Sporadic	Few or no first- or second-degree relatives affected by cancer	Low
	Cancer occurs in only one generation	
	There is no particular pattern of inheritance	
	Later age of cancer onset	

The second phase of the study was conducted five years later. The families that were previously identified as having at least one first-degree and one second-degree relative affected by cancer were visited again, and were re-interviewed by one of the former research assistants (LCLJr.) to confirm the previously reported CFH. The informant interviewed in phase 2 was not necessarily the same person who responded to the questionnaire in phase 1 of the study. All participants were previously contacted by telephone to schedule the interview. At the beginning of the interview, the researcher clarified that the family was contacted again because of its risk to present a familial susceptibility to cancer. Noteworthy, neither the researcher nor the participant had access to the pedigree that was depicted at the first visit. The same questions that were asked in the first interview were asked again. This study was approved by the Institutional Review Board (No.215/CEP/CSE-FMRP-USP).

## Results

**Figure 1:**
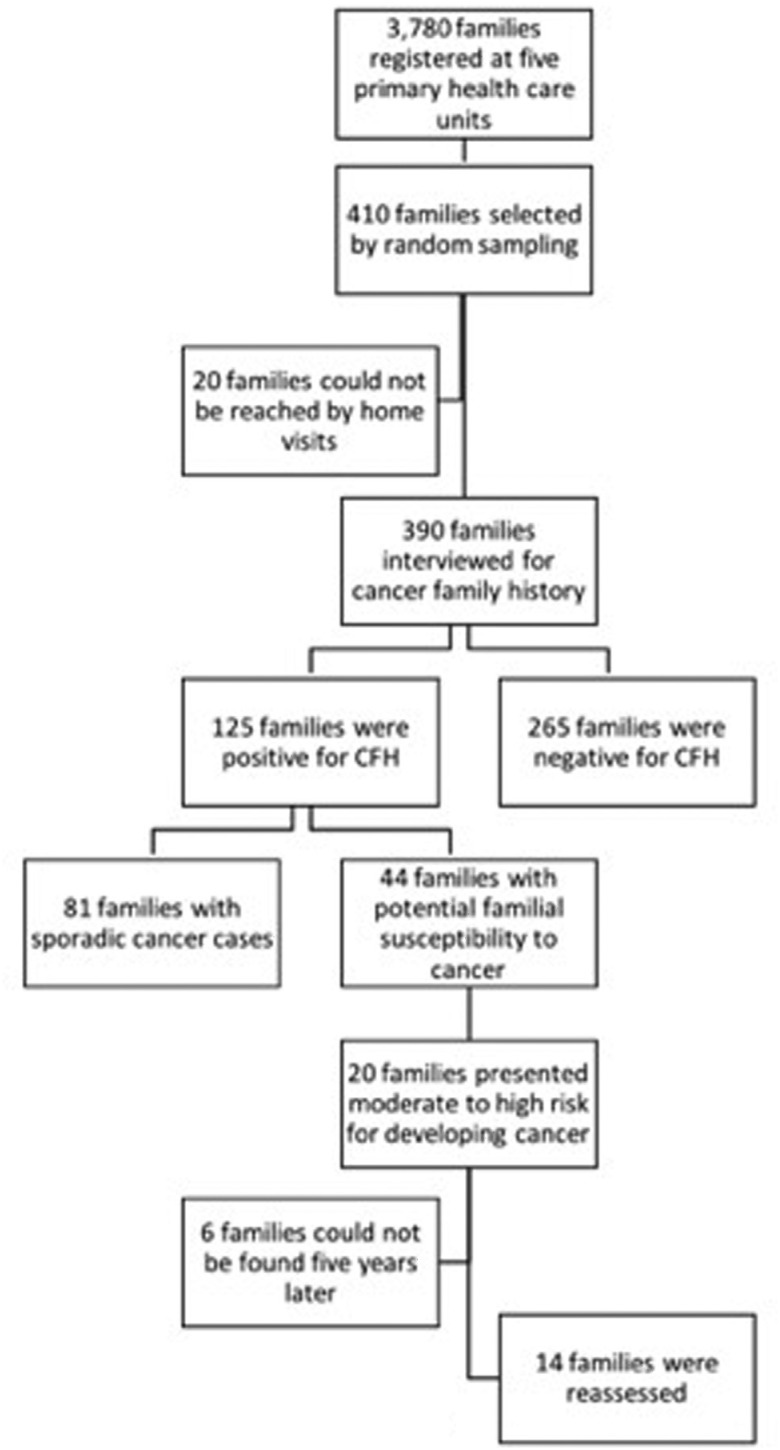
Study flowchart including the number of evaluated families which relevant information were obtained in each step of the data collection process.

Of the 3,780 families registered in five primary health care units, 410 were randomly chosen for this study. Our final sample was composed of 390 families with which personal contact was possible. The characteristics of their pedigrees are shown in Table 2. Most participants were female (79.5%), and sample mean age was 54.0 ± 16.5 years. Informative familial history - namely, complete information on the occurrence of cancer (and when present, tumor site and age at onset of disease) for at least three generations - was obtained from 219 (56.1%) families. Of the 390 families interviewed, 125 (32.0%) informed to be affected by cancer (Figure 1): in 20 (29.4%) families at least one first- and one second-degree relative of the respondent were affected; in 14 (24.6%) at least one first-degree relative of the respondent was affected; in 48 (70.6%) only second-degree and more distant relatives were affected; and 43 (75.4%) had only more distant family members with cancer. Also, 10 respondents had had cancer themselves.

**Table 2:** Characterization of family members according to their age, number, and gender of individuals in the pedigrees.

Variables	Mean ± SD (range)
Age of respondents, y	54.0 (16.5) 18-95
Age of family members affected by cancer, y	57.5 (13.2) 20-92
Age of youngest family member affected by cancer, y	50.3 (17.1) 3-92
Number of generations	3.2 (0.76) 2-6
Number of family members	16.0 (7.0) 3-40
Number of female family members	6.9 (3.5) 2-16
Number of male family members	9.1 (5.0) 2-30
Number of studied families	390

SD, standard deviation; y, years.

Based on the analysis of relatedness, age of disease onset, primary tumor sites and clusters, as determined by internationally established criteria for familial cancer susceptibility syndromes, we identified 81 (64.8%) families with sporadic cancer cases, and 44 (35.2% within the families affected by cancer, or 11.3% of the overall study sample) with potential familial susceptibility to cancer. Regarding the hereditary cancer syndromes, we found four families that met the criteria for breast and ovary hereditary cancer, three for Li-Fraumeni syndrome, two families with syndromes that comprised hereditary colorectal cancer, another two families with breast and colon cancer, three families with potential alterations on repair genes, one family probably carrier of hereditary gastric cancer syndrome, and others with familial cancer clusters without a specific inheritance pattern.

Respondents reported cases of breast (n = 29), prostate (n = 29), pelvic (n = 16), colorectal (n = 14), and hematologic (n = 12) cancers, as well as melanoma (n = 6), tumors that are commonly associated with hereditary cancer syndromes (Table 3). Importantly, these results point to the existence of previously unidentified families (11.3% of the overall studied sample healthy population) potentially at risk for familial susceptibility to cancer.

**Table 3:** Distribution of malignant neoplasms according to the casuistry of cancer in the affected families.

	Frequency (n)			Total
Sites of malignant neoplasms	Sporadic	Familial	Potentially hereditary	Neoplasms
Head and neck	31	10	6	47
Lung	15	8	7	30
Breast	13	9	7	29
Prostate	11	5	13	29
Stomach	5	9	8	22
Pelvic	2	7	7	16
Colorectal	4	5	5	14
Womb	8	5	1	14
Liver	7	2	4	13
Hematological	4	1	7	12
Non-melanoma skin	4	6	1	11
Melanoma	2	2	2	6
Bone	5	1	0	6
Pancreas	3	0	0	3
Esophagus	1	1	1	3
Ovary	1	0	0	1
Unknown	8	2	10	20
Others	7	7	14	28
Total	131	80	93	304

We next investigated whether self-reported family history is sufficiently consistent for use in genetic risk estimation for inherited cancer. For this, five years later we revaluated 14/20 families that we previously identified as having at least one first- and one second-degree relative affected by cancer and presenting moderate (n = 4) to high (n = 16) risk for developing cancer based on genetic predisposition (Figure 1). Analysis of family history collected in the second interview confirmed 90% of initial pedigrees, regardless of whether the interviewed family member was the same subject (n = 11) from the first interview or not (n = 3). In addition, we observed new cancer red flags in these families, such as benign lesions at early ages and new cancer cases that reinforce the family's high risk for hereditary cancer syndromes (Lindor *et al.*, 2008).

## Discussion

Recognizing patterns of familial cancer that indicate increased risk and possible hereditary syndromes can help to identify individuals who may benefit from preventive interventions (Ashton-Prolla, 2013). In this study we used self-reported family history data to examine the risk of hereditary cancer syndromes among middle- and low-income families registered at primary care centers in Brazil. We also revaluated the cancer family history of families at risk five years later to determine the consistency of the information collected through the use of spontaneous self-reports.

Our results showed that among those families affected by cancer, 35.2% had moderate to high risk of familial susceptibility to cancer. This represents a relatively high prevalence of potential hereditary cancer syndromes in the study sample. Likewise, a significant prevalence (6.2%) of hereditary breast cancer syndromes have been reported in a previous study in which CFH was assessed in women from primary health care units of an underserved region in southern Brazil (Palmero *et al.*, 2009). These results point to the likely - yet often neglected - benefits of screening policies aimed at identifying individuals at risk in these settings (Doerr and Teng, 2012; Teng and Acheson, 2014). A pioneering initiative to identify individuals with an increased risk for hereditary breast cancer syndromes was conducted through the successfully development and validation of a simple questionnaire, which is sensitive and specific in primary care setting, as a screening tool to refer at-risk individuals for genetic counseling (Ashton-Prolla *et al.*, 2009).

Given that cancer family history is a dynamic measure that changes significantly over time, its periodic reassessment has been recommended (Lu *et al.*, 2014; Wood *et al.*, 2014). Upon revaluation, we found that cancer family history was confirmed, and expanded, for 90% of the families interviewed. In some cases, we collected data regarding benign lesions that are often found on the clinical spectrum of the hereditary cancer syndromes (Lindor *et al.*, 2008; Lu *et al.*, 2014; NCCN, 2014). These data point to the reliability of self-reports as a means of early identification of healthy individuals at risk of cancer. Indeed, literature data have shown that self-reports of personal cancer history are generally reliable, especially for breast, prostate, and colon cancer (Roth *et al.*, 2009; Scheuner *et al.*, 2010). Most sensitivity values for self-reports of a positive family history of cancer in a first-degree relative range from 70 to 90% (Ziogas and Anton-Culver, 2003; Murff *et al.*, 2007). Our study demonstrated that, even after a long period of time, self-reported CFH is an effective mean to detect not only breast, prostate, and colon cancer, but other important tumors that are considered as part of the spectrum of the hereditary cancer syndromes, such as Li-Fraumeni syndrome, which has shown considerable prevalence in the Brazilian population (Achatz *et al.*, 2007; Giacomazzi *et al.*, 2013).

The inclusion of new health technologies, including genetic risk assessment and genetic testing, remains a challenge for middle-income countries like Brazil and other Latin American countries (Palmero *et al.*, 2009). Therefore, the possibility to use self-reports to unveil familial susceptibility to cancer, as revealed here, may be particularly beneficial in those settings characterized by a lack of electronic health records or individual health history. Notably, no population-based notification system exists in the Primary Health Care Brazilian database (Vieira *et al.*, 2015). It should be emphasized that none of the 20 families that fulfilled the established criteria for familial cancer susceptibility syndromes, and that were identified as having moderate to high risk to hereditary cancer syndromes (Lindor *et al.*, 2008; Valdez *et al.*, 2010; NCCN, 2014; Vieira *et al.*, 2015), had their CFH registered in their medical records at the community-based primary care centers where they were followed.

Although cancer management has been predominantly focused on the individuals affected by disease, not on their families, our results indicate that better management may be achieved by including family screening practices in primary care policies. Therefore, to enhance family practices and adherence to health policies, primary care work force should receive training and ongoing education, which should include essential competencies to collect family history, such as, basic genetics and genomics knowledge, communication skills, ability to establish empathetic interpersonal relationships, and capacity to deal with relevant ethical issues (Flória-Santos *et al.*, 2013).

Even though barriers to incorporating family history taking and hereditary risk assessment in middle-income primary care settings do exist, the wider use of self-reported cancer family history can be a useful tool to achieve this goal.

## References

[B1] Achatz MI, Olivier M, Le Calvez F, Martel-Planche G, Lopes A, Rossi BM, Ashton-Prolla P, Giugliani R, Palmero EI, Vargas FR (2007). The TP53 mutation, R337H, is associated with Li-Fraumeni and
Li-Fraumeni-like syndromes in Brazilian families. Cancer Lett.

[B2] Ashton-Prolla P (2013). Hereditary cancer syndromes: Opportunities and
challenges. BMC Proc.

[B3] Ashton-Prolla P, Giacomazzi J, Schmidt AV, Roth FL, Palmero EI, Kalakun L, Aguiar ES, Moreira SM, Batassini E, Belo-Reyes V (2009). Development and validation of a simple questionnaire for the
identification of hereditary breast cancer in primary care. BMC Cancer.

[B4] Doerr M, Teng K (2012). Family history: Still relevant in the genomics era. Cleve Clin J Med.

[B5] Feerro WG, Bigley MB, Brinner KM, The Family, Health History Multi-Stakeholder Workgroup of the American Health
Information Community (2008). New standards and enhanced utility for family health history
information in the electronic health record: An update from the American Health
Information Community's Family Health History Multi-Stakeholder
Workgroup. J Am Med Inform Assoc.

[B6] Floria-Santos M, Santos EMM, Nascimento LC, Pereira-da-Silva G, Ferreira BR, Miranda DO, Lopes LC, Pinto PS (2013). Oncology nursing practice from the perspective of genetics and
genomics. Text Context Nursing.

[B7] Giacomazzi J, Selistre SG, Rossi C, Alemar B, Santos-Silva P, Pereira FS, Netto CB, Cossio SL, Roth DE, Brunetto AL (2013). Li-Fraumeni and Li-Fraumeni-like syndrome among children diagnosed
with pediatric cancer in Southern Brazil. Cancer.

[B8] Gomy I, Diz MDPE (2013). Hereditary cancer risk assessment: Essential tools for a better
approach. Hered Cancer Clin Pract.

[B9] Janssens AC, Henneman L, Detmar SB, Khoury MJ, Steyerberg EW, Eijkemans MJ, Mushkudiani N, Oostra BA, van Duijn CM, Mackenbach JP (2012). Accuracy of self-reported family history is strongly influenced by the
accuracy of self-reported personal health status of relatives. J Clin Epidemiol.

[B10] Kelly KM, Shedlosky-Shoemaker R, Porter K, Remy A, DeSimone P, Andrykowski MA (2007). Cancer family history reporting: Impact of method and psychosocial
factors. J Genet Couns.

[B11] Lindor NM, McMaster ML, Lindor CJ, Greene MH, National Cancer Institute Division of Cancer Prevention and Community
Oncology and Prevention Trials Research Group (2008). Concise handbook of familial cancer susceptibility syndromes. Second
edition. J Natl Cancer Inst Monogr.

[B12] Lu KH, Wood ME, Daniels M, Burke C, Ford J, Kauff ND, Kohlmann W, Lindor NM, Mulvey TM, Robinson L (2014). American society of clinical oncology expert statement: Collection and
use of a cancer family history for oncology providers. J Clin Oncol.

[B13] Murff HJ, Greevy R, Syngal S (2007). The comprehensiveness of family cancer history assessments in primary
care. Community Genet.

[B14] Pagano M, Gauvreau K (2004). Principles of Biostatistics.

[B15] Palmero EI, Caleffi M, Schüler-Faccini L, Roth FL, Kalakun L, Netto CBO, Skonieski G, Giacomazzi J, Weber B, Giugliani R (2009). Population prevalence of hereditary breast cancer phenotypes and
implementation of a genetic cancer risk assessment program in southern
Brazil. Genet Mol Biol.

[B16] Plat AW, Kroon AA, Van Schayck CP, De Leeuw PW, Stoffers HE (2009). Obtaining the family history for common, multifactorial diseases by
family physicians. A descriptive systematic review. Eur J Gen Pract.

[B17] Qureshi N, Carroll JC, Wilson B, Santaguida P, Allanson J, Brouwers M, Raina P (2009). The current state of cancer family history collection tools in primary
care: A systematic review. Genet Med.

[B18] Roth FL, Camey SA, Caleffi M, Schuler-Faccini L, Palmero EI, Bochi C, Moreira SM, Kalakun L, Giugliani R, Ashton-Prolla P (2009). Consistency of self-reported first-degree family history of cancer in
a population-based study. Fam Cancer.

[B19] Rubinstein WS, Acheson LS, O’Neill SM, Ruffin MT, Wang C, Beaumont JL, Rothrock N, Family Healthware Impact Trial Group (2011). Clinical utility of family history for cancer screening and referral
in primary care: A report from the Family Healthware impact trial. Genet Med.

[B20] Scheuner MT, McNeel TS, Freedman AN (2010). Population prevalence of familial cancer and common hereditary cancer
syndromes. The 2005 California health interview survey. Genet Med.

[B21] Schneider K (2002). Counseling About Cancer: Strategies for Genetic Counselors.

[B22] Teng K, Acheson LS (2014). Genomics in primary care practice. Prim Care.

[B23] Valdez R, Yoon PW, Qureshi N, Green RF, Khoury MJ (2010). Family history in public health practice: A genomic tool for disease
prevention and health promotion. Annu Rev Public Health.

[B24] Vieira DK, Attianezi M, Esposito AC, Barth A, Sequeira C, Krause N, Oliveira V, Lucidi A, Serao C, Llerena JC (2015). Identification of familial clustering for cancer through the family
health strategy program in the municipality of Angra dos Reis, Rio de Janeiro,
Brazil. J Community Genet.

[B25] Weitzel JN, Blazer KR, Mac Donald DJ, Culver OJ, Offit K (2011). Genetics, genomics and risk assessment: State of the art and future
directions in the era of personalized medicine. CA Cancer J Clin.

[B26] Wood ME, Kadlubek P, Pham TH, Wollins DS, Lu KH, Weitzel JN, Neuss MN, Hughes KS (2014). Quality of cancer family history and referral for genetic counseling
and testing among oncology practices: A pilot test of quality measures as part of
the American Society of Clinical Oncology Quality Oncology Practice
Initiative. J Clin Oncol.

[B27] Ziogas A, Anton-Culver H (2003). Validation of family history data in cancer family
registries. Am J Prev Med.

